# Air-oxidation from sulfur to sulfone-bridged Schiff-base macrocyclic complexes showing enhanced antimicrobial activities

**DOI:** 10.1038/s41598-017-15898-1

**Published:** 2017-11-21

**Authors:** Genfeng Feng, Yunshan Shi, Lei Zhang, Rongguang Shi, Wei Huang, Ruiyong Wang

**Affiliations:** 1 State Key Laboratory of Coordination Chemistry, Nanjing National Laboratory of Microstructures, School of Chemistry and Chemical Engineering, Nanjing University, Nanjing, 210093 P. R. China; 2 State Key Laboratory of Pharmaceutical Biotechnology, School of Life Science, Nanjing University, Nanjing, 210093 P. R. China

## Abstract

Two embedded sulfur atoms in a novel [2 + 2] Schiff-base macrocyclic dinuclear Zn(II) complex were found to be easily autoxidized to the sulfone units on air exposure, and the resultant sulfone-functionalized macrocyclic complex was obtained by the post-modification strategy exhibiting enhanced antimicrobial activities because of the presence of dual active sites in comparison with the sulfur-containing Schiff-base macrocycle.

## Introduction

Macrocyclic molecules have attracted considerable and increasing attention because of the mainstay role they play in supramolecular chemistry^1–5^, biochemistry^6–8^, material science^9,10^, separation^11,12^, encapsulation^13–15^ and catalysis^16,17^. Among them, Schiff-base macrocycles occupy a special status owing to their facile syntheses from the Schiff-base condensation and versatile coordination fashions with metal ions. On the other hand, heteroatom-functionalized macrocycles also represent one challenging research topic due to the difficulties in tuning the ring size, configuration and binding modes^18–21^. The incorporation of heteroatoms into Schiff-base macrocycles is often achieved by using easily accessible heteroatom-containing diamines and triamines, such as diethylenetriamine, 1,2-bis(2-aminoethoxy)ethane and tris(2-aminoethyl)amine^22–25^. In contrast, the attempts to design and synthesize extended dialdehydes are believed as another impressive method to yield heteroatom-functionalized Schiff-base macrocycles^22,26–30^.

**Figure 1:**
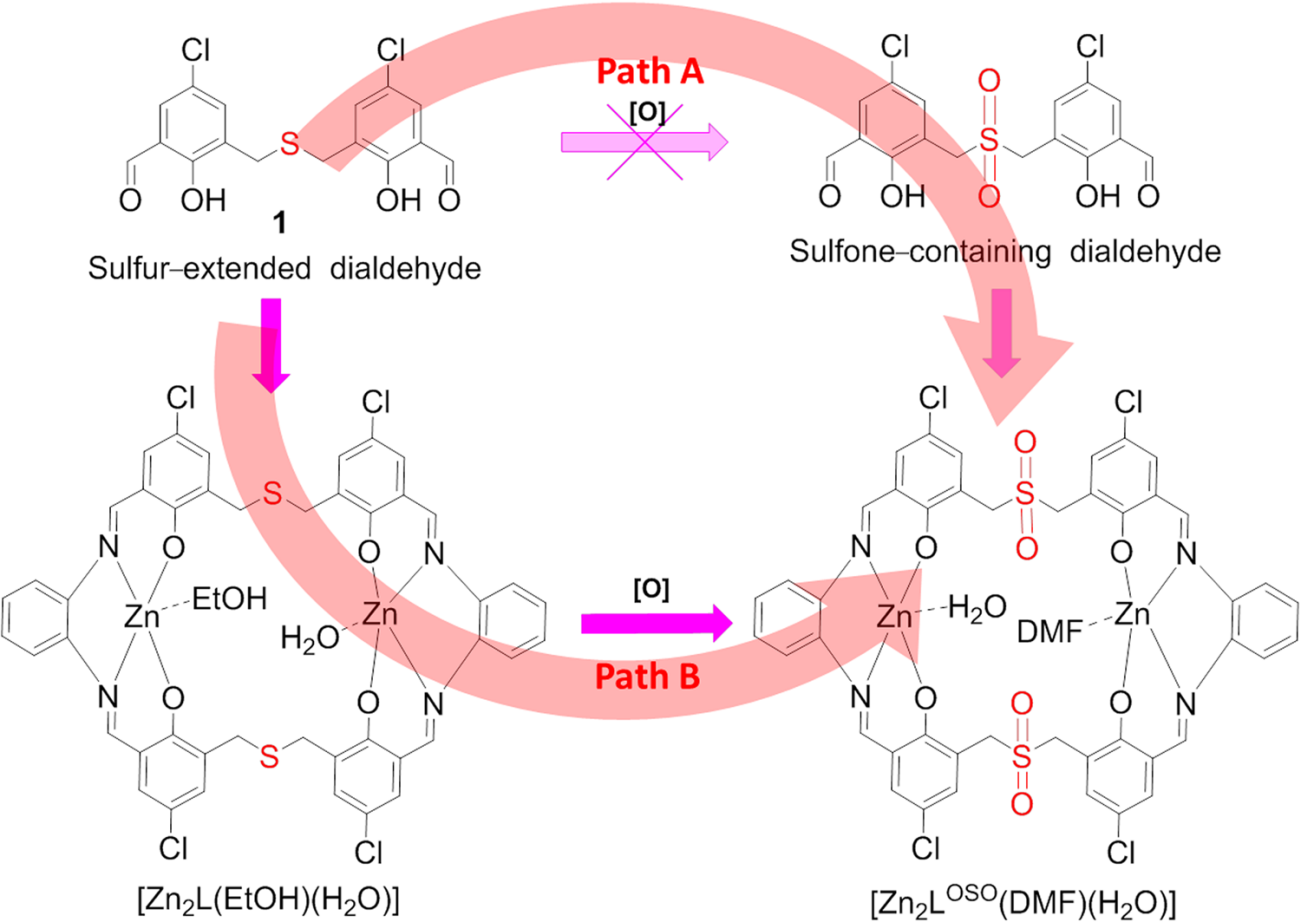
Synthetic illustration of macrocyclic Zn(II) complexes.

However, sulfur-bridged Schiff-base macrocycles especially the flexible ones are rarely reported up till now^31–38^. It is known that thiacalixarenes are an important subclass of calixarenes and they can be readily oxidized into the forms of sulfoxides and even sulfones by post-oxidation process displaying improved properties than corresponding thiacalixarenes^39–41^. This post-modification is regarded as a powerful synthetic strategy for finely tuning functionalities of resultant products. In this work, we intend to introduce the sulfur atoms into the Schiff-base macrocyclic skeletons and implant the post-modification synthetic strategy of thiacalixarenes to generate the sulfur-containing Schiff-base macrocycles and corresponding oxidized products. Thus, a new sulfur-extended dialdehyde **1** (Fig. 1) was prepared via the nucleophilic substitution between 5-chloro-3-(chloromethyl)-2-hydroxybenzaldehyde and sodium sulfide and a [2 + 2] sulfur-bridged Schiff-base dinuclear macrocyclic Zn(II) complex [Zn_2_L]^42^ was produced by the template-assisted synthesis. Furthermore, The sulfone-functionalized [2 + 2] Schiff-base macrocyclic Zn(II) complex [Zn_2_L^OSO^] was obtained successfully via post-oxidation of [Zn_2_L].

## Results and Discussion

Different from thiacalixarenes having two oxidized forms, only one type of oxidized product [Zn_2_L^OSO^] could be detected by ESI-MS (Figure S1) and isolated in a high yield of 67.8% from the H_2_O_2_ oxidation of [Zn_2_L]. The difference may be ascribed to the presence of aliphatic sulfur ether moieties and coordinated metal ions in [Zn_2_L] compared with the metal free aromatic sulphide in thiacalixarenes. Actually, the air was found to be adequate in this oxidized process because [Zn_2_L^OSO^] was first obtained as crystals in the process of growing single crystals under ambient condition. However, H_2_O_2_ was used in a control experiment to accelerate the oxidation process and H_2_O_2_ was proved to be a more effective reagent to produce [Zn_2_L^OSO^]. The good consistency of the sulfone-product obtained from air and H_2_O_2_ oxidation was verified by corresponding ^1^H NMR spectra. (Figure S2). In addition, we intended to prepare [Zn_2_L^OSO^] through the direct Schiff-base condensation between sulfone-containing dialdehyde and 1,2-diaminobenzene (Path A in Scheme 1), but sulfur-extended dialdehyde **1** showed no selectivity under H_2_O_2_ oxidation and sulfone-containing dialdehyde could not be isolated (Figures S3 and S4). The reason is that aldehyde, phenolic hydroxyl and sulfur units in compound **1** are all sensitive to the oxidant. That is to say, it is invalid to access the targeting [Zn_2_L^OSO^] via Path A. In contrast, only path B is available for preparing macrocycle [Zn_2_L^OSO^] where the two uncoordinated sulfur atoms are selectively oxidized to sulfone groups, because the easily oxidized aldehyde groups in **1** have been transformed into the imine connected macrocycle and stabilized by the following Zn(II) ion complexation in [Zn_2_L]. In addition, another oxygen-unstable phenolic hydroxyl groups have been deactivated via electron delocalization originated from the formation of coordinative bonds with Zn(II) ions. So it is concluded that the post-modification synthetic strategy is particularly useful in preparing sulfone-functionalized Schiff-base macrocycle in our case.

**Figure 2:**
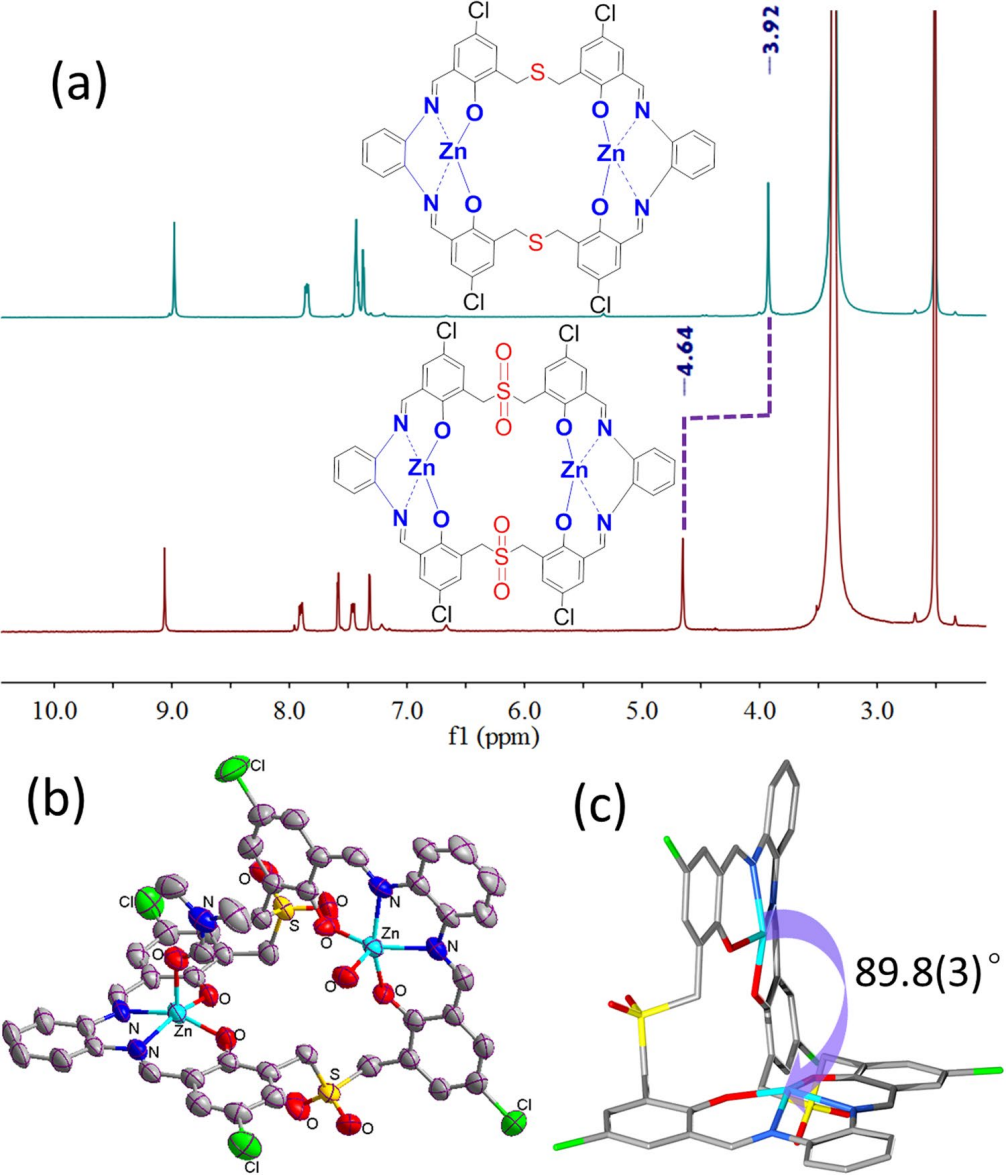
(**a**) ^1^H NMR spectral comparisons of dried macrocyclic Zn(II) complexes [Zn_2_L] and [Zn_2_L^OSO^] in DMSO-*d*_6_; (**b**) ORTEP drawing of [Zn_2_L^OSO^(DMF)(H_2_O)] at the 50% probability level; (**c**) View of the dihedral angle between the two N_2_O_2_ Salen coordination planes in [Zn_2_L^OSO^(DMF)(H_2_O)], where the apical ligands are omitted for clarity.

FT-IR, ESI-MS and ^1^H NMR spectra were performed to verify the two types of macrocyclic complexes. A medium FT-IR absorption peak was observed at 1649 cm^−1^ in the dialdehyde precursor, indicative of the presence of aldehyde groups (Figure S5). However, a new absorption peak was found in the sulfur-bridged macrocyclic complex (1615 cm^−1^) indicating the transformation from the aldehyde groups to Schiff-base C=N units (Figure S6). ESI-MS of [Zn_2_L] revealed one positive peak at *m/z* = 1075.00 corresponding to {[Zn_2_L] + EtOH + H_2_O + H}^+^ (Figure S7). The successful generation of sulfone-based complex was evidenced by the observation of a new positive peak at *m/z* = 1121.25 in [Zn_2_L^OSO^] assigned as {[Zn_2_L^OSO^] + EtOH + H}^+^ specie (Figure S1) by contrast. Furthermore, ^1^H NMR spectral comparisons were carried out to verify the changes in chemical shifts between the sulfur and sulfone bridged macrocyclic complexes. As depicted in Fig. 2a, the chemical shift of methylene protons (*δ* = 3.92 ppm) adjacent to the sulfur atoms in [Zn_2_L] is significantly shifted to the lower field (*δ* = 4.64 ppm) in [Zn_2_L^OSO^], because of the stronger deshielding effects of sulfone groups. In addition, the FT-IR spectrum of sulfone-functionalized macrocyclic complex manifests the disappearance of aldehyde groups and the emergence of imine moieties (Figure S8).

X-ray single-crystal diffraction analysis of the sulfur-bridged dialdehyde precursor reveals a folded structure in which the two phenolic rings have a dihedral angle of 15.2(1)°. Intramolecular π-π stacking interactions are present between them with the centroid-to-centroid separation of 3.675(2) Å. Furthermore, intermolecular π-π stacking interactions are found between neighboring molecules with the longer centroid-to-centroid distance of 3.708(2) Å forming an infinite supramolecular chain (Figure S9). In comparison with dialdehyde precursor **1**, the two phenolic rings are expanded in macrocycle [Zn_2_L^OSO^(DMF)(H_2_O)] accompanied by the destruction of intramolecular π-π stacking. The tetra Schiff-base macrocyclic complex [Zn_2_L^OSO^(DMF)(H_2_O)] has a [2 + 2] dinuclear structure (Fig. 2b), and the two Zn(II) centers have similar square-pyramidal coordination geometry with slightly different *τ* values of 0.043 and 0.064, respectively^43^. The basal plane is composed of two imine nitrogen atoms and two phenolic oxygen atoms, and the apical position for each is occupied by a water molecule and a N,N-dimethylformamide molecule, respectively. The related Zn-O and Zn-N bond lengths fall within the normal range of 1.944(3) ~ 2.092(4) Å. Different from the conventional *µ*_2_ bridging mode for each phenolic oxygen atom, the two N_2_O_2_ Salen coordination planes in our 30-membered macrocyclic Zn(II) complex are essentially perpendicular with a bite angle of 89.8(3)° (Fig. 2c), and the distance between two Zn(II) centers is measured as 6.977(3) Å.

**Figure 3:**
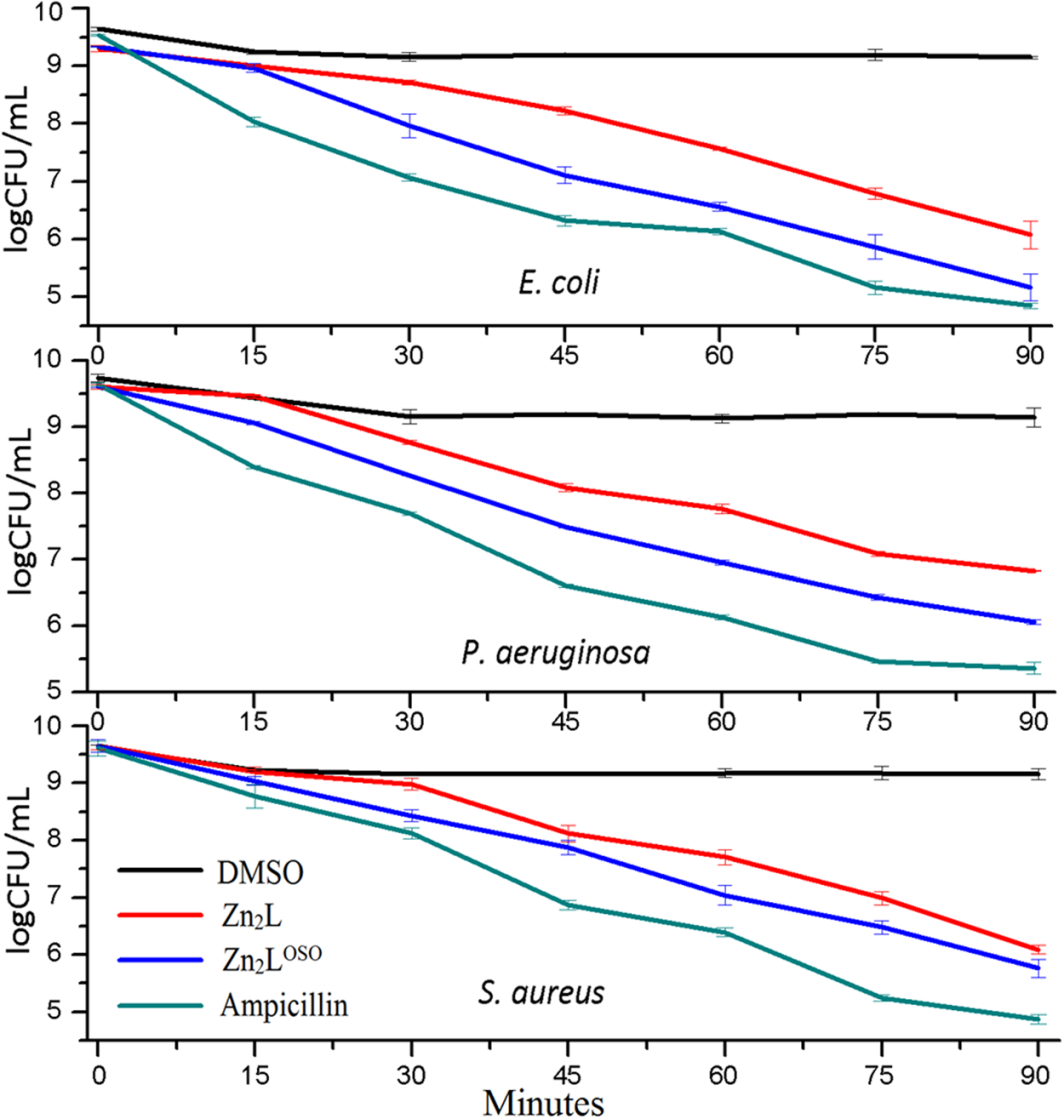
Time-kill assay of three bacteria after the treatment with two macrocyclic complexes over 1.5 h.

Considering sulfones and Schiff-base macrocycles are both antibacterially active^44–48^, enhanced antibacterial activities are expected for our dual-functional macrocyclic complex [Zn_2_L^OSO^]. In the following work, antimicrobial experiments of [Zn_2_L^OSO^] have been carried out for *E*. *coli* (ATCC 2567), *P*. *aeruginosa* (ATCC 2036) and *S*. *aureus* (ATCC 2079) together with [Zn_2_L] for comparison. The preliminary antimicrobial activities are assessed by determining their MIC (minimum inhibitory concentration) and MBC (minimum bactericidal concentration) values. As can be seen in Table 1, both [Zn_2_L] and [Zn_2_L^OSO^] show significantly lower MIC and MBC values than dialdehyde **1**. Compared to the reference compound ampicillin, the results of two macrocyclic complexes are lower, but undoubted improvement of MIC and MBC values can be observed from [Zn_2_L] to [Zn_2_L^OSO^]. Moreover, the enhanced antimicrobial activities could be confirmed through a time-kill assay over 1.5 h, together with ampicillin as a standard and DMSO as the blank. In contrast to ampicillin, both sulfone-functionalized and sulfur-bridged macrocycles exhibit less effective antimicrobial activities against all the three bacterial strains along with time prolonged, but the former displays more obvious decrease of logCFU/mL than the latter (Fig. 3). It is suggested that the enhanced antimicrobial activities from macrocycle [Zn_2_L] to [Zn_2_L^OSO^] could be ascribed to the incorporation of two bioactive sites into one molecule in our case.

**Table 1:**
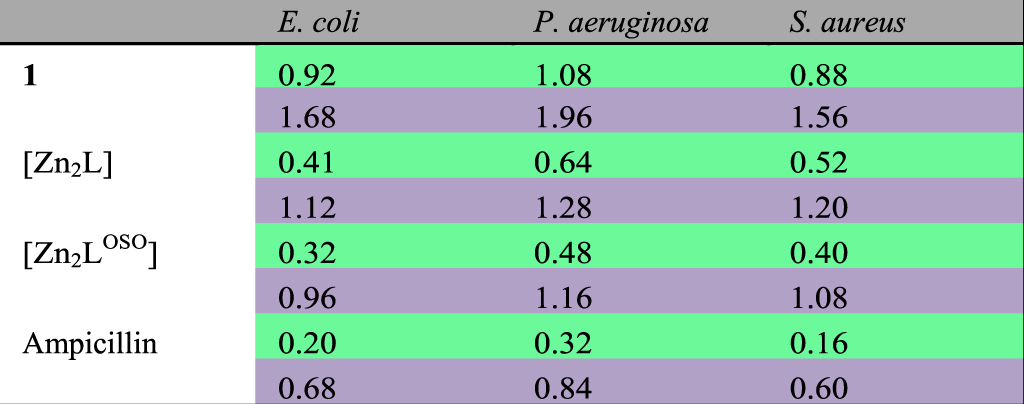
Antibacterial activities of the related compounds against three kinds of bacteria. The antibacterial activities are expressed as the MIC and MBC. The data in the first line for every compound (green) represent MIC and those in the second line (purple) stand for MBC. The unit of all value is mg/mL.

## Conclusions

**Figure 4:**
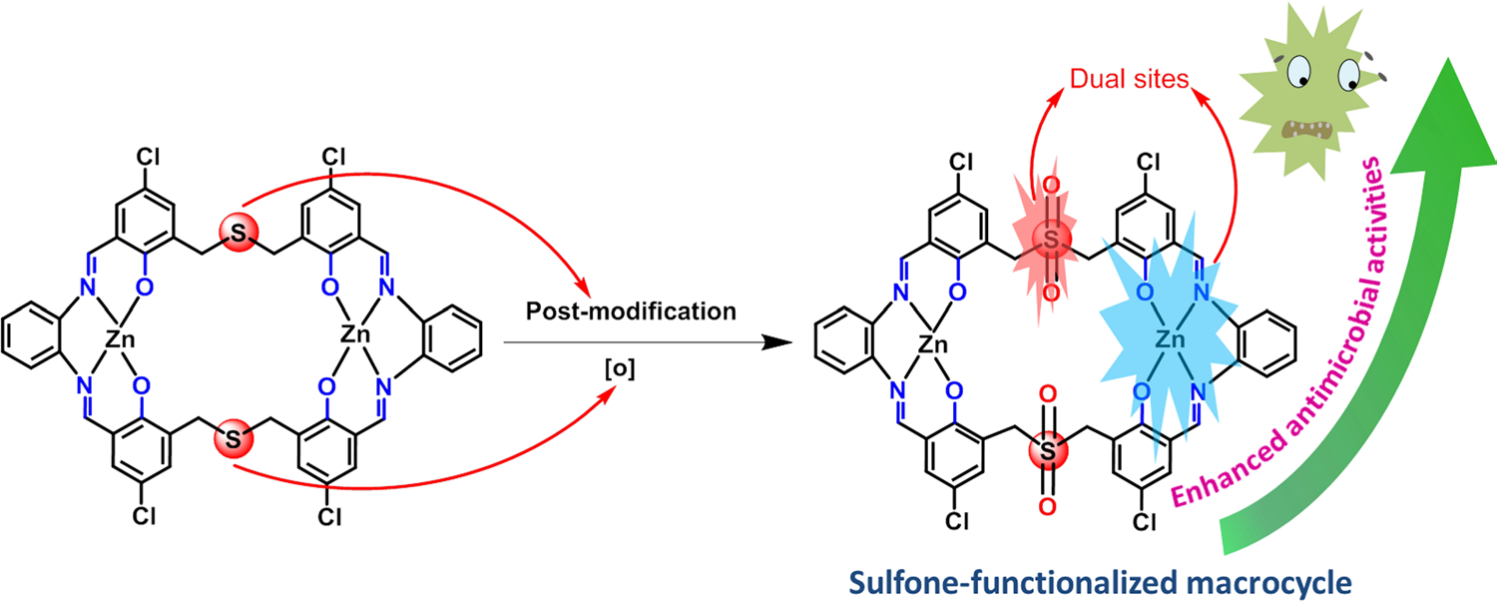
Schematic illustration for the air-oxidation from sulfur to sulfone-bridged Schiff-base macrocyclic complexes with dual bioactive sites displaying enhanced antimicrobial activities.

In summary, a novel [2 + 2] sulfur-bridged Schiff-base macrocyclic dinuclear Zn(II) complex was synthesized from a sulfur-extended dialdehyde via the Schiff-base condensation, and the sulfur atoms in the macrocyclic skeleton were found to be facilely *in situ* oxidized to the sulfone units even in ambient condition. It is noted that this effective post-modification from sulfur to sulfone after forming the macrocyclic complex is necessary because the phenolic hydroxyl and aldehyde units in the dialdehyde precursor are both sensitive to the oxidant. More interestingly, the sulfone-functionalized Schiff-base macrocyclic complex with dual bioactive sites displays enhanced antimicrobial activities in contrast to the undecorated macrocycle (Fig. 4). Considering the scarcity of flexible Schiff-base macrocyclic backbones with sulfur-containing bridges, the current study is believed to provide a practical approach to access multi-functional macrocyclic molecules by means of the post-modification strategy.

## Electronic supplementary material

Supplementary Information
