# Supplementary material for: Air-oxidation from sulfur to sulfone-bridged Schiff-base macrocyclic complexes showing enhanced antimicrobial activities
Source: Sci Rep. 2017 Nov 21;7:15881. doi: 10.1038/s41598-017-15898-1 (PMC5698483; doi:10.1038/s41598-017-15898-1)
Supplement: Supplementary file 1 — Supplementary Information [file 41598_2017_15898_MOESM1_ESM.pdf]

Electronic Supplementary Information

Air-oxidation from sulfur to sulfone-bridged Schiff-base macrocyclic complexes showing enhanced antimicrobial activities

Genfeng Feng,[a] Yunshan Shi,[b] Lei Zhang,[a] Rongguang Shi,[a] Wei Huang*[a] and Ruiyong Wang*[b]

[a]*State Key Laboratory of Coordination Chemistry, Nanjing National Laboratory of Microstructures, School of Chemistry and Chemical Engineering, Nanjing University, Nanjing, 210093, P. R. China.*
[b]*State Key Laboratory of Pharmaceutical Biotechnology, School of Life Science, Nanjing University, Nanjing 210093, P. R. China.*

S1 General

The reagents of analytical grade were purchased from commercial sources and used without any further purification. Infrared spectra (4000−400 cm^{-1}) were recorded using a Nicolet FT-IR 170X spectrophotometer at 25 ℃ using KBr plates. ^1H NMR spectra were obtained in a Bruker 400 MHz NMR spectrometer. Electrospray ionization mass spectra (ESI-MS) were recorded on a ThermoFisher Scientific LCQ Fleet mass spectrometer. Elemental analyses were performed on a PerkinElmer 240 analyzer.

S2 Synthesis

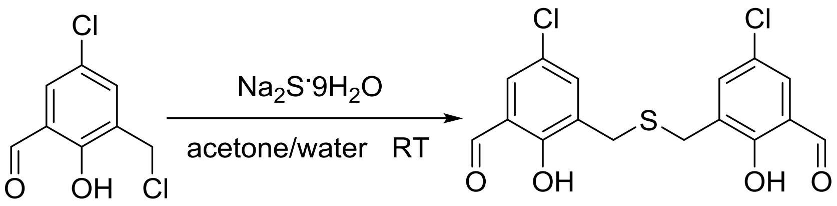

Scheme S1. Synthesis of sulfur-extended dialdehyde **1**.

Sulfur-extended dialdehyde 1: 5-Chloro-3-(chloromethyl)-2-hydroxybenzaldehyde was synthesized by our previously reported method.[1] $Na_2S·9H_2O$ (0.96 g, 4.0 mmol) was dissolved in the mixture of acetone and water (40 mL, v/v = 5:1). An acetone solution (20 mL) of 5-chloro-3-(chloromethyl)-2-hydroxybenzaldehyde (1.64 g, 8.0 mmol) was added dropwise, and the mixture was stirred for 3 h at room temperature. The yellow solid was purified by silica gel column chromatography (petroleum ether/ethyl acetate/acetic acid, v/v/v = 10:1:0.1) after removing the solvents under reduced pressure. The final pure sulfur-extended dialdehyde **1** was obtained in a yield of 0.92 g (62.2 %). Main FT-IR absorptions (KBr pellets): 2925 (m), 1649 (m), 1433 (m), 1282 (m), 1205 (m), 999 (m), 717 (m). ^1H NMR (400 MHz, $CDCl_3$, ppm): δ = 11.30 (s, 2H, -OH), 9.83 (s, 2H, -CHO), 7.49 (s, 2H, Ph-H), 7.41 (s, 2H, Ph-H), 3.75 (s, 4H, $-CH_2-$). ESI-MS in methanol (negative mode): Calcd $C_{16}H_{12}Cl_2O_4S$, $[M-H]^-$: 368.98; Found: 369.00.

Complex [Zn_2L]: A solution of $Zn(NO_3)_2$ $6H_2O$ (32.7 mg, 0.11 mmol) dissolved in 10 mL EtOH was added into a solution of dialdehyde 1 (37.1 mg, 0.10 mmol) in a mixture of hot EtOH (10 mL) and DMF (10 mL). The mixture was refluxed for 10 min, and a solution of 1,2-diaminobenzene (10.8 mg, 0.10 mmol) in EtOH (10 mL) was added. The yellowish solution was stirred for another 3 h, cooled to RT, and filtered. The filtrate was concentrated to give complex **2** in a yield of 72.9 % (39.2 mg). The solid of complex **2** was thoroughly dried in vacuo to remove all the solvent molecules for the following characterizations. ^1H NMR (400 MHz, DMSO-d_6, ppm): δ = 8.97 (s, 4H, CH=N), 7.85 (s, 4H, Ph-H), 7.36-7.43 (m, 12H, Ph-H), 3.92 (s, 8H, -CH_2-). ^{13}C NMR (101 MHz, DMSO-d_6, ppm): δ = 168.26, 162.36, 139.35, 133.21, 133.03, 127.73, 119.51, 116.84, 115.07, 30.95. Main FT-IR absorptions (KBr pellets): 3402 (m), 1615 (s), 1533 (s), 1448 (m), 1199 (m), 751 (m). Elemental analysis: calcd (%) for $C_{44}H_{28}Cl_4N_4O_4S_2Zn_2$: C 52.15, H 2.79, N 5.33; found (%): C 52.08, H 2.85, N 5.42.

Complex [Zn_2L^{OSO}]:

Route I (via air oxidation). After completing the above-mentioned Schiff-base reaction between the sulfur-bridged dialdehyde and 1,2-diaminobenzene under Zn(II) template effect, the solution was filtered and the filtrate was evaporated slowly at room temperature in air for two weeks. And light-yellow single crystals of [Zn_2L^{OSO}(DMF)(H_2O)] were obtained directly.

Route II (via H_2O_2 oxidation). After completing the above-mentioned Schiff-base reaction between the sulfur-bridged dialdehyde and 1,2-diaminobenzene under Zn(II) template effect, excess 30 % H_2O_2 (50.0 mg, 0.44 mmol) was added and stirred overnight. The solution was filtered and the filtrate was concentrated to give the sulfone-functionalized [2+2] Schiff-base macrocyclic Zn(II) complex in 67.8 % yield (38.1 mg). The solid of sulfone-functionalized macrocyclic complex was thoroughly dried in vacuo to remove all the solvent molecules for the following characterizations. ^1H NMR (400 MHz, DMSO-d_6, ppm): δ = 9.05 (s, 4H, CH=N), 7.88-7.90 (m, 4H,

Ph-H), 7.57 (s, 4H, Ph-H), 7.44-7.46 (m, 4H, Ph-H), 7.31 (s, 4H, Ph-H), 4.64 (s, 8H, -CH_2-). Main FT-IR absorptions (KBr pellets): 3409 (m), 1619 (s), 1535 (s), 1387 (m), 1201 (m), 1124 (m). Elemental analysis: calcd (%) for $C_{44}H_{28}Cl_4N_4O_8S_2Zn_2$: C 49.05, H 2.62, N 5.20; found (%): C 48.96, H 2.75, N 5.31.

Control experiment of the dialdehyde oxidation: Sulfur-extended dialdehyde (0.20 g, 0.54 mmol) was dissolved in ethanol, and then equivalent 30 % H_2O_2 (61.2 mg, 0.54 mmol) was added. After the stirring for 6 h at room temperature, ^{1}H NMR and ESI-MS spectra were used to monitor the reaction. The results indicated the disappear of H signals corresponding to the aldehyde and phenolic hydroxyl units in the dialdehyde precursor (Figure S3) as well as a neglectable peak at *m/z* = 369.00 in ESI-MS (Figure S4), but the expected oxidation product could not be identified from both spectra.

S3 X-ray crystallographic analysis

X-ray single-crystal diffraction data for dialdehyde precursor **1** and macrocycle [Zn_2L^{OSO}(DMF)(H_2O)] were measured on a Bruker SMART 1K CCD diffractometer using graphite monochromatic Mo Kα radiation (λ = 0.71073 Å). Data collection was performed by using the SMART program and cell refinement and data reduction were made with the SAINT program.[2] The crystal system was determined by Laue symmetry and the space groups were assigned on the basis of systematic absences by using XPREP. The structures were solved by the directed method and refined on F^2 by using the full-matrix least-squares methods with SHELXTL version 6.10.[3] All non-hydrogen atoms were refined on F^2 by full-matrix least-squares procedure using anisotropic displacement parameters. Hydrogen atoms were inserted in the calculated positions assigned fixed isotropic thermal parameters at 1.2 times the equivalent isotropic U of the atoms to which they are attached (1.5 times for the oxygen atoms)

and allowed to ride on their respective parent atoms. In the case of $[Zn_2L^{OSO}(DMF)(H_2O)]$, the SQUEEZE program implemented in PLATON was used to remove the highly disordered guest molecules because no satisfactory disorder model could be achieved, where an A type error was generated when checked by PLATON (VERY LARGE Solvent Accessible VOID(S) in Structure). In addition, it was difficult for us to collect the high-quality diffraction data for $[Zn_2L^{OSO}(DMF)(H_2O)]$, in which a B type error was generated when checked by PLATON (Number of (Iobs-Icalc)/SigmaW > 10 Outliers). However, we think the structural mode of $[Zn_2L^{OSO}(DMF)(H_2O)]$ is reasonable and the refinement results are reliable. All calculations were carried out with the SHELXTL PC program package. Details of the data collection and refinement are given in Table S1

Table S1. Crystal data and structure refinement

Compound	**1**	**3**·3DMF
Empirical formula	$C_{16}H_{12}Cl_2O_4S$	$C_{56}H_{58}Cl_4N_8O_{13}S_2Zn_2$
Formula weight	371.22	1387.76
Temperature	291(2) K	291(2) K
Wavelength	0.71073 Å	0.71073 Å
Crystal system	monoclinic	triclinic
Space group	$P2_1/n$	$P\bar{1}$
a	8.475(1) Å	12.179(2) Å
b	13.963(1) Å	16.274(3) Å
c	13.502(1) Å	20.415(3) Å
α	90 °	106.082(5) °
β	100.125(1) °	105.116(5) °
γ	90 °	102.614(5) °
Volume	1573.0(2) $Å^3$	3564.5(10) $Å^3$
Z	4	2
Density (calculated)	1.568 g/cm^3	1.293 g/cm^3
Absorption coefficient	0.562 mm^{-1}	0.940 mm^{-1}
F(000)	760	1428
Crystal size	0.12 x 0.10 x 0.10 mm^3	0.12 x 0.10 x 0.10 mm^3

θ range for data collection	2.84 to 27.59 °	2.04 to 25.00 °
Index ranges	$-7 \le h \le 11$, $-17 \le k \le 18$, $-17 \le l \le 17$	$-13 \le h \le 14$, $-19 \le k \le 18$, $-23 \le l \le 24$
Reflections collected	10571	25472
Independent reflections	3634 [R(int) = 0.0188]	12460 [R(int) = 0.0338]
Completeness	99.9 %	99.1 %
Max. and min. transmission	0.946 and 0.936	0.912 and 0.896
Refinement method	Full-matrix least-squares on F^2	Full-matrix least-squares on F^2
Data / restraints/ parameters	3634 / 0 / 210	12460 / 0 / 774
GOF (Goodness-of-fit) on F^2	1.044	1.048
Final R indices [I > 2sigma(I)]	R_1 = 0.0320, wR_2 = 0.0874	R_1 = 0.0497, wR_2 = 0.1541
R indices (all data)	R_1 = 0.0384, wR_2 = 0.0921	R_1 = 0.0760, wR_2 = 0.1659
Largest diff. peak and hole	0.240 and -0.283 e·Å^{-3}	0.431 and -0.310 e·Å^{-3}

$R_1 = \Sigma||Fo|-|Fc||/\Sigma|Fo|$; $wR_2 = [\Sigma[w(Fo^2-Fc^2)^2]/\Sigma w(Fo^2)^2]^{1/2}$, $w = 1/[\sigma^2(Fo^2) + (0.0448P)^2] + 0.4800P$ in **1** and $w = 1/[\sigma^2(Fo^2) + (0.0969P)^2]$ in **3**·3DMF, where $P = (Fo^2 + 2Fc^2)/3)$.

S4 Antimicrobial activity

Determination of minimum inhibitory and bactericidal concentrations: The antibacterial activities of related compounds were studied against *E. coli* (ATCC 2567), *S. aureus* (ATCC 2079) and *P. aeruginosa* (ATCC 2036) bacteria. Aliquots of samples were serially diluted in NB (nutrient broth) medium in 96 well plates to produce a concentration range of 2-76 μg/mL. The final concentration of microorganisms was adjusted to 5×10^{-5} CFU/mL. Plates were incubated at 36 ℃ for 24 h and the MICs were determined as the lowest concentration of additive that demonstrated no visible growth. For MBCs determination, 100 μL of the broth from wells without visible growth were plated onto NA (Nutrient Agar) and were incubated for 24 h at 36 ℃. The MBCs were defined as the lowest concentration that could kill 99.9 % of bacterial population.

Time-kill assay: The antimicrobial activity comparison was further studied using a time-kill assay by measuring the reduction in the numbers of CFU per milliliter of bacteria (*E. coli*, *S. aureus* and *P. aeruginosa*) over 1.5 h. Briefly, the bacterial suspension (0.5 to 0.6 cm^{-1}) was exposed to the test agent at 1×MIC, and incubated at 37 ℃. As for ampicillin, the MIC value of sulfone macrocycle was chosen as the

experimental concentration. After 0, 15, 30, 45, 60, 75 and 90 min of incubation, aliquots of each sample were diluted and plated onto NA, and then the viable counts were determined after 24 h of incubation at 36 ℃. Killing curves were constructed by plotting the logCFU/mL versus time.

S5. Additional Figures

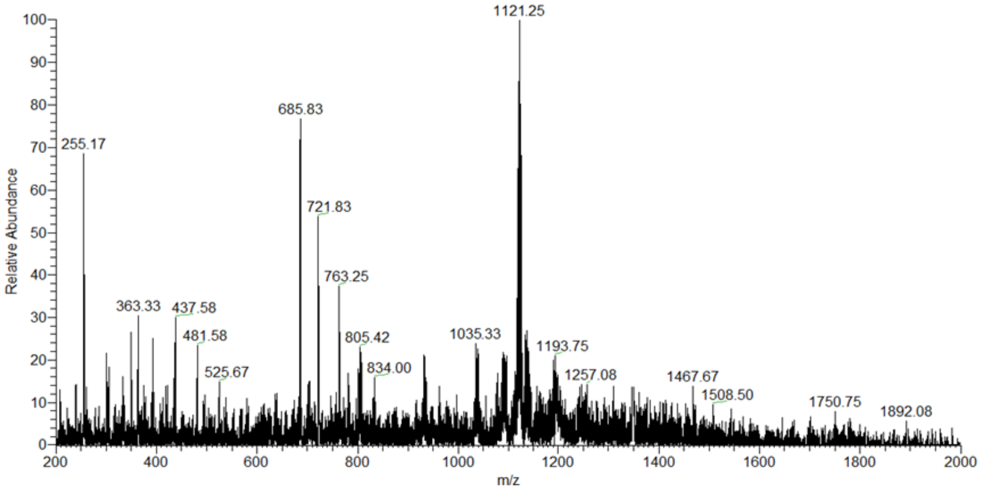

Figure S1. ESI-MS of [Zn_2L^{OSO}] (positive mode).

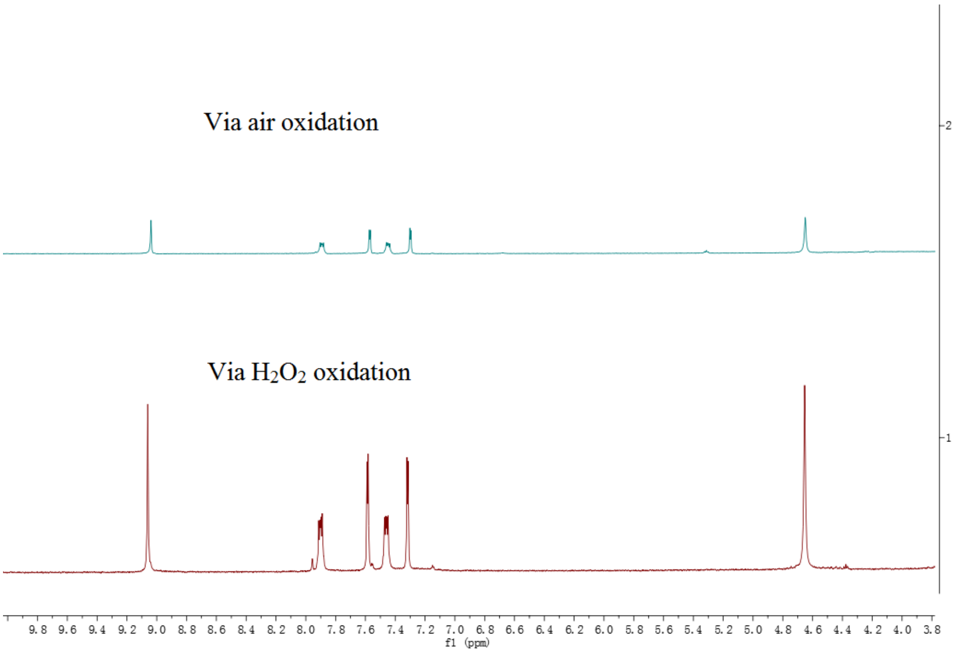

Figure S2. ^{1}H NMR comparison of two oxidation regents (air and H_2O_2)

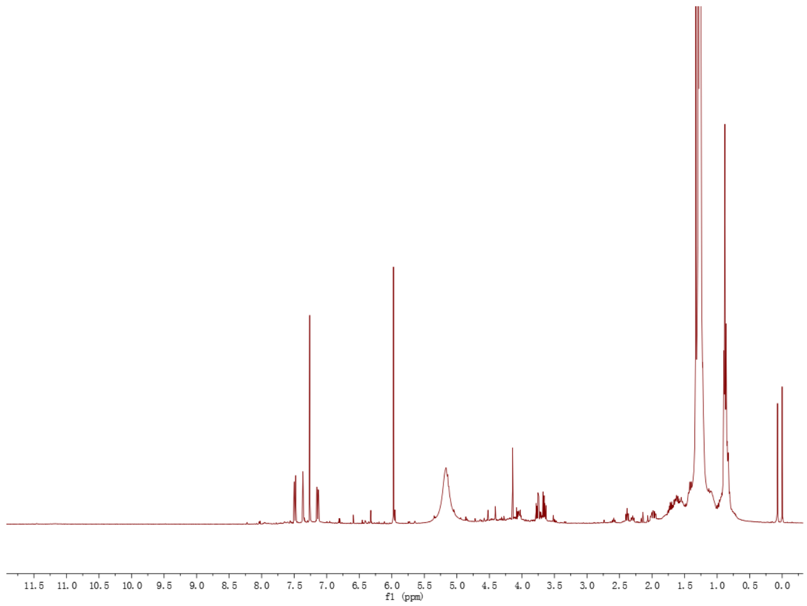

Figure S3. ^{1}H NMR spectrum of sulfur-bridged dialdehyde after adding H_2O_2.

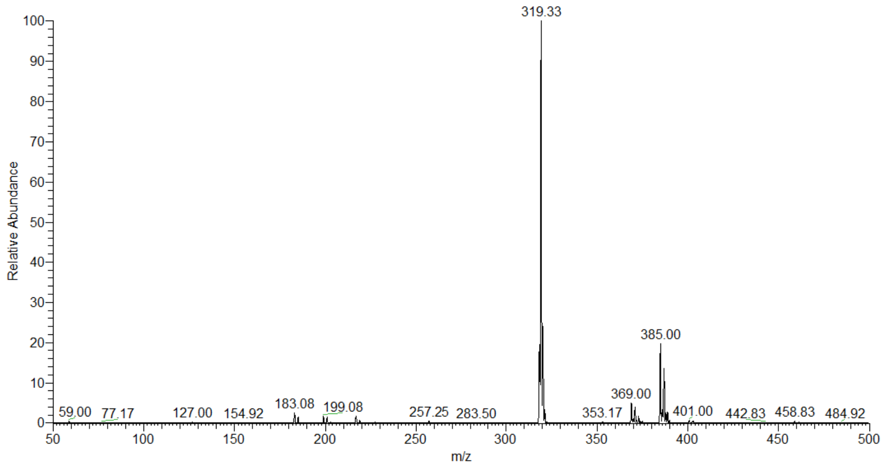

Figure S4. Negative mode ESI-MS of the sulfur-bridged dialdehyde after adding H_2O_2.

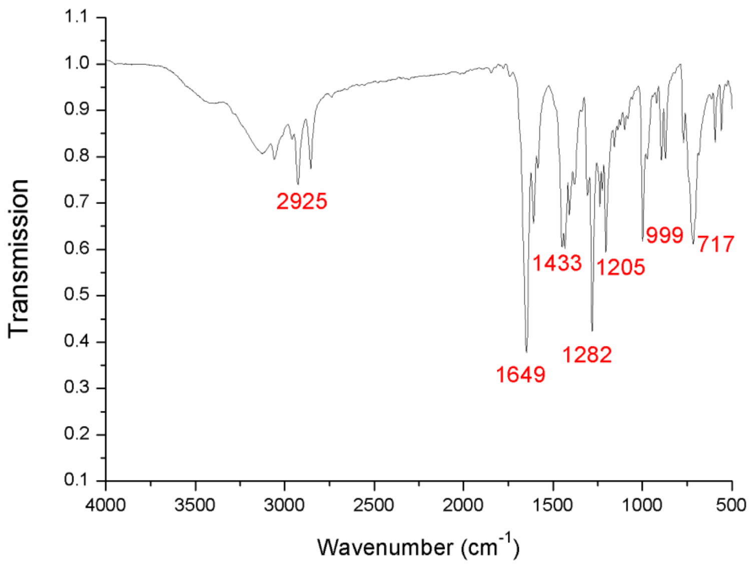

Figure S5. FT-IR spectrum of sulfur-extended dialdehyde **1**.

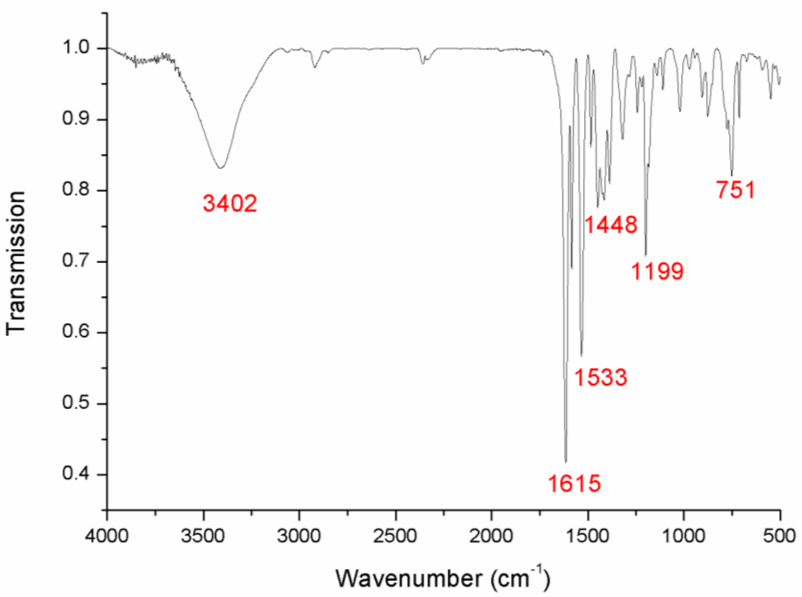

Figure S6. FT-IR spectrum of [Zn_2L].

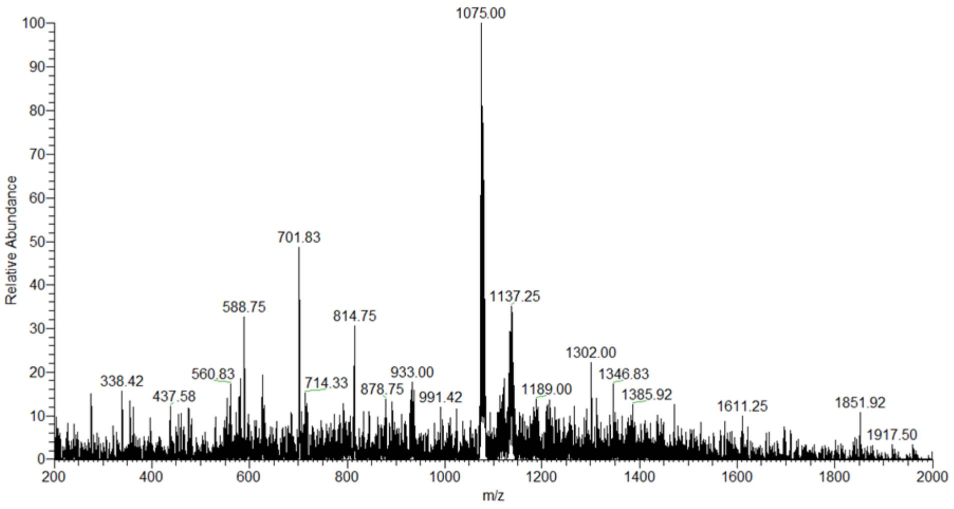

Figure S7. ESI-MS of $[Zn_2L]$ (positive mode).

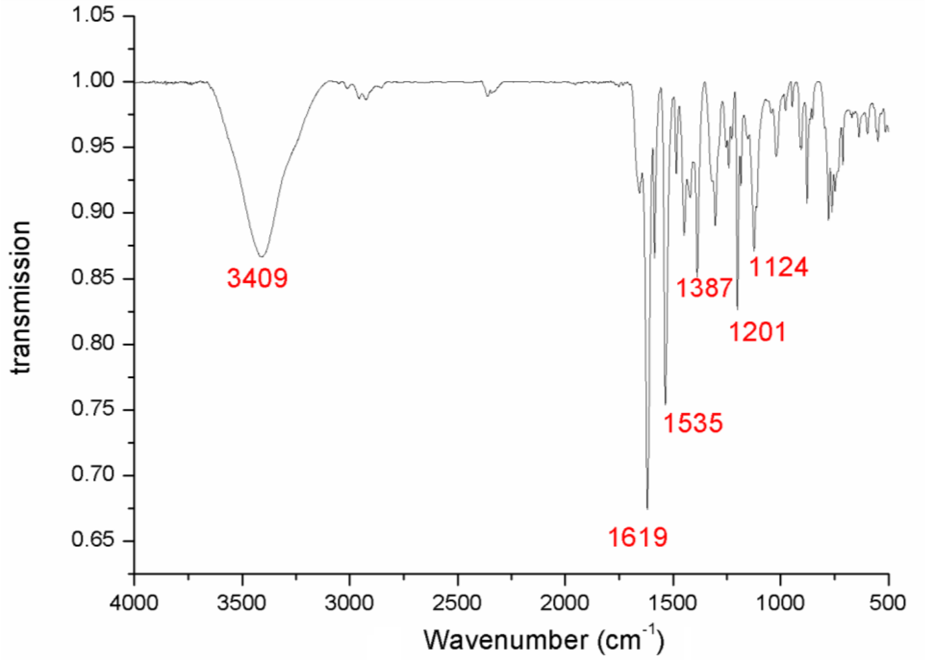

Figure S8. FT-IR spectrum of $[Zn_2L^{OSO}]$.

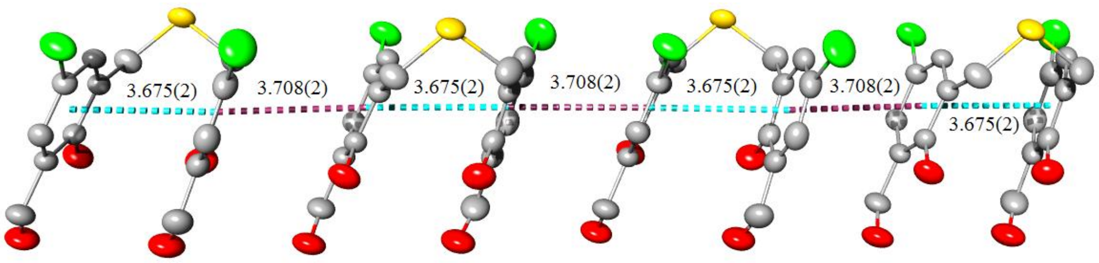

Figure S9. Infinite one-dimensional chain of dialdehyde precursor sustained by π-π stacking interactions.

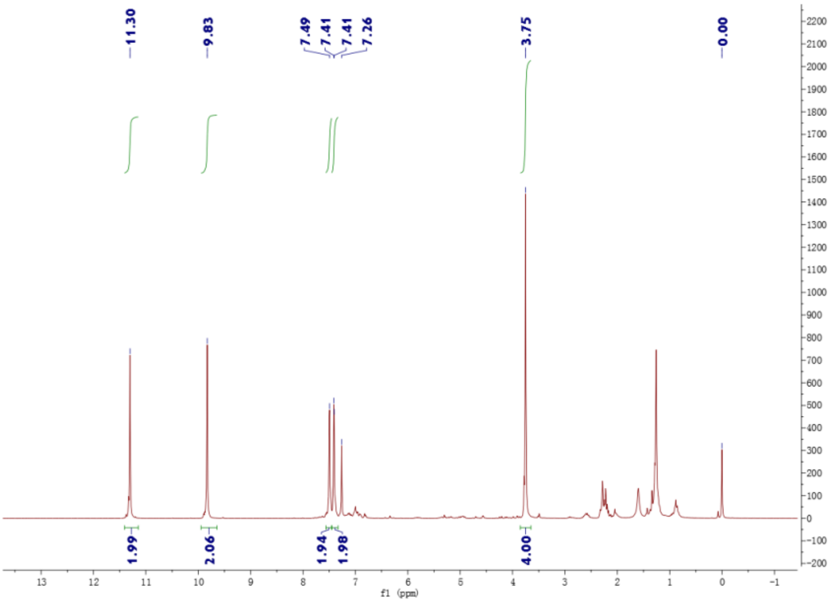

Figure S10. ^{1}H NMR spectrum of sulfur-extended dialdehyde **1.**

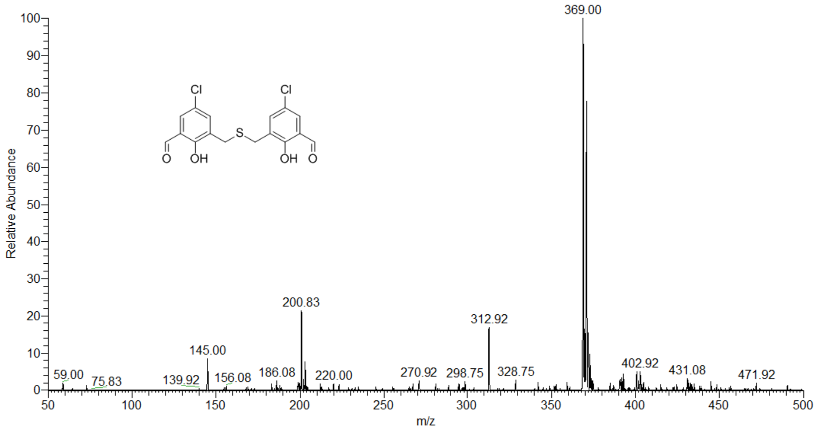

Figure S11. ESI-MS of sulfur-extended dialdehyde **1** (negative mode).

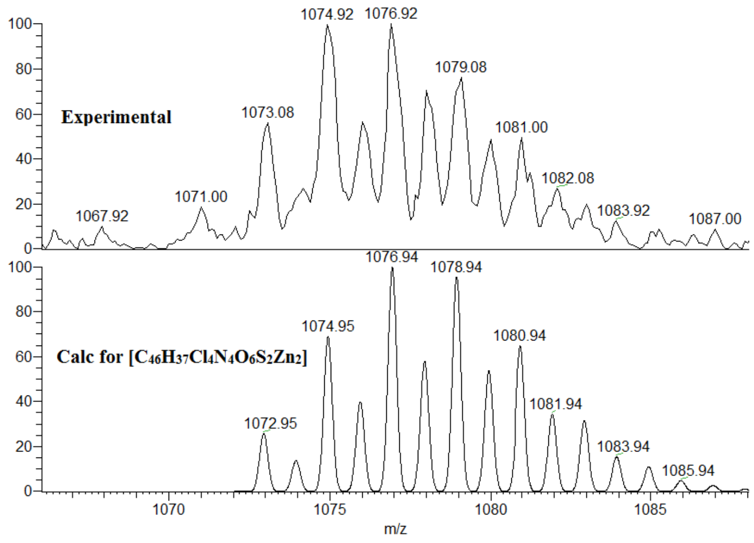

Figure 12. The experimental and calculated isotopic distribution corresponding to the peak at *m/z* 1075 with 100 % abundance in [Zn_2L].

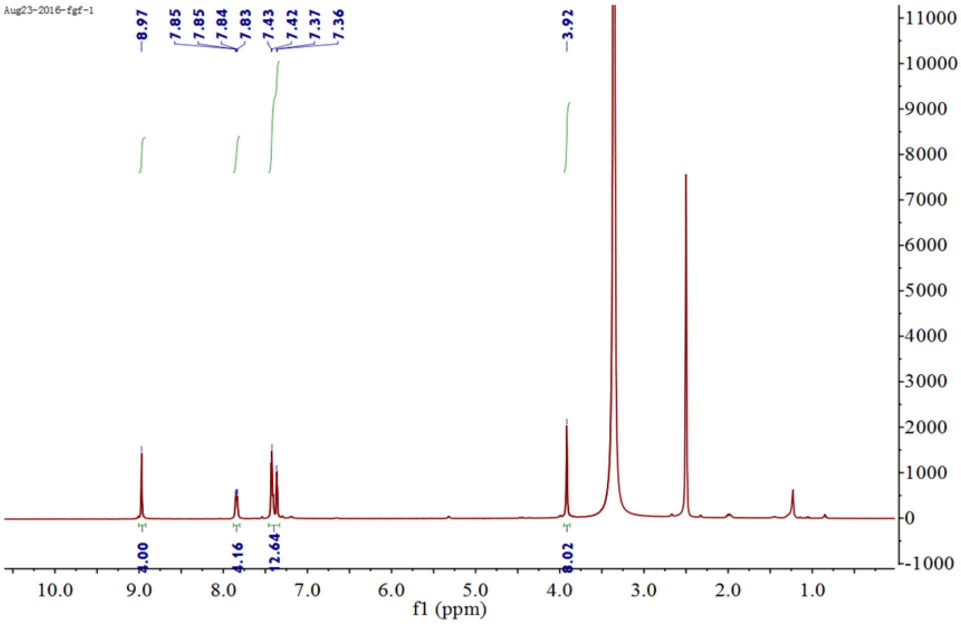

Figure S13. ^{1}H NMR spectrum of [Zn_2L].

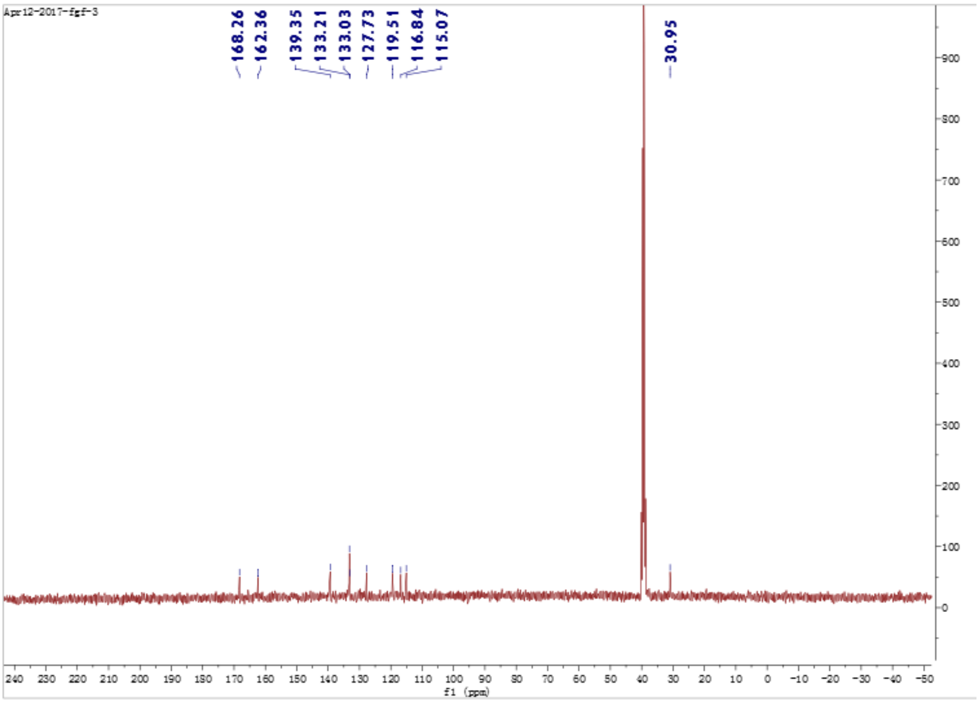

Figure S14. ^{13}C NMR spectrum of [Zn_2L].

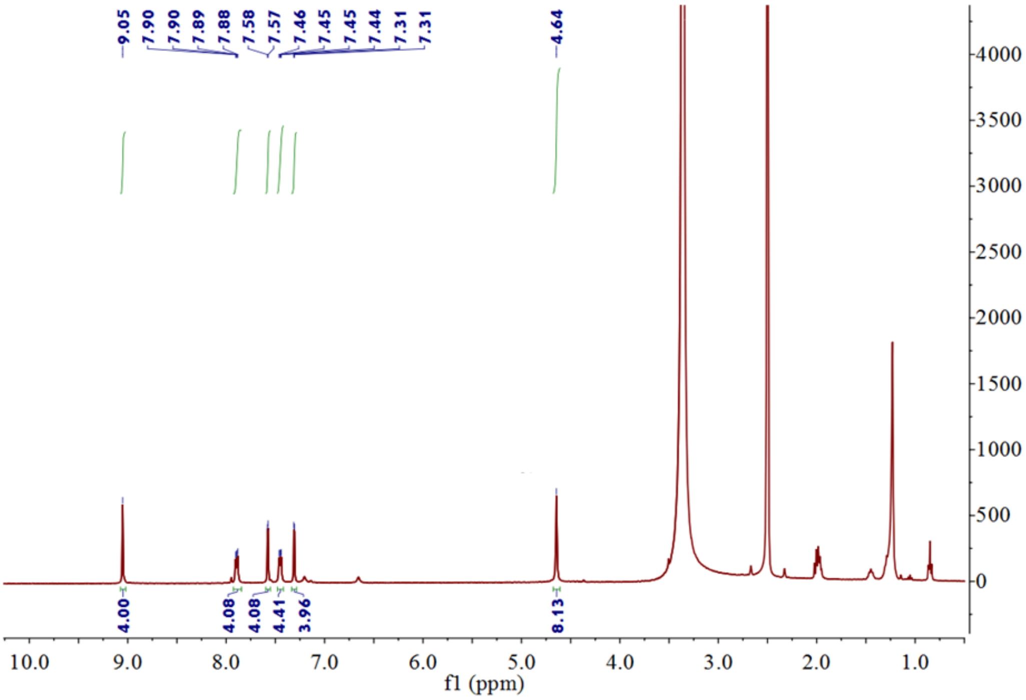

Figure S15. ^{1}H NMR spectrum of [Zn_2L^{OSO}].

S6. References

1 Huang, W. *et al*. An improved oxidation approach for preparing 4-substituted-2, 6-diformylphenol by manganese (IV) dioxide. *Synth. Commun.* **30**, 1555–1561 (2000).

2 Sheldrick, G. M. SADABS, Program for Empirical Absorption Correction of Area Dectector Data, Univ. of Gottingen, Germany, 2000.
3 Siemens, SHELXTL, Version 6.10 Reference Manual, Siemens Analytical X-Ray Systems, Inc., Madison, Wisconsin, USA, 2000.
